# Pulmonary function in relation to muscle endurance and functional capacity in children with bronchial asthma

**DOI:** 10.1186/s12890-025-03650-9

**Published:** 2025-04-29

**Authors:** Zahera Raafat Zedan, Walaa Abd El-Hakiem Abd El-Nabie, Shimaa AboEldahab Ahmed, Eman Mohamed Tantawy, Mai Mohamed Khalaf

**Affiliations:** 1 Department of Physical Therapy for Pediatrics, Benha University, Banha, Egypt; 2 Department of Physical Therapy for Pediatrics, Cairo University, Giza, Egypt; 3 Chest Diseases Consultant, Abbaseya Chest Hospital, Cairo, Egypt; 4 Department of Pediatrics, Faculty of Physical Therapy, Benha University, Banha, Egypt

**Keywords:** Bronchial asthma, Pulmonary functions, Muscle endurance, And functional capacity

## Abstract

**Background:**

Bronchial asthma is a major global health concern among children. It poses a significant global public challenge, causing around 22.8 million years of life lost to disability and 495,100 asthma-related deaths.

**Objective:**

The goal of the study was to investigate the relationship between pulmonary function in children with bronchial asthma and both the muscle endurance of the deep cervical flexors and overall functional capacity.

**Methods:**

This cross-sectional study involved sixty-four pediatric patients diagnosed with bronchial asthma, aged from 8 to 10 years old from both sexes. Their body mass index was from 5th percentile to less than 85th percentile for age, gender and height. They were evaluated and diagnosed by using spirometry. Pulmonary function (vital capacity(VC), peak expiratory flow (PEF), forced expiratory volume in 1s (FEV1), forced expiratory volume in 1s / forced vital capacity (FEV1/FVC), muscle endurance, and functional capacity were assessed by using spirometry, pressure biofeedback, and 6-minutewalk test respectively.

**Results:**

The findings indicated a clear and significant positive association between pulmonary function measures, including (VC, PEF, FEV1 and the FEV1/FVC ratio), with both the cervical flexion test and the six-minute walk test (6MWT). These correlations were statistically significant, with *p*-values of ≤ 0.05.

**Conclusion:**

Pulmonary function is associated with endurance of cervical flexors and functional capacity in pediatric patients with bronchial asthma.

## Introduction

Bronchial asthma is a long-lasting inflammatory condition marked by fluctuating airway blockage, resulting in heightened sensitivity, inflammation, and respiratory issues. It is common among children and a significant cause of hospital admissions in this age group globally. While various asthma phenotypes exist and predictive indicators are difficult to establish, certain clinical risk factors have been recognized. These include having atopic dermatitis, a family history of asthma, elevated total immunoglobulin E (IgE) levels (over 60 kU/l), and exposure to tobacco smoke [1].

Pulmonary function tests (PFTs) allow physicians to evaluate their patients’ respiratory function across different clinical situations, especially when there are contributing elements related to respiratory conditions, workplace exposures, or pulmonary harm. The accuracy of PFTs results depends on the patient’s effort. While PFTs do not give a definitive diagnosis, their findings should be analyzed in conjunction with the patient’s medical history, physical assessment, and laboratory results to assist in determining the patient’s condition. Additionally, PFTs help physicians measure the severity of lung disease, track its progression over time, and assess the effectiveness of treatments [2].

According to the Global Initiative for Asthma (GINA) guidelines, when diagnosing asthma, clinicians should document the following: (A) The presence of multiple respiratory symptoms that are often worse at night or early in the morning, varying in intensity and over time, and can be triggered by colds, exercise, exposure to allergens, laughter, or smoke; (B) Confirmation of expiratory airflow limitations, indicated by a reduced FEV1 and a decreased FEV1/FVC ratio (which is typically greater than 0.90 in healthy children); (C) Evidence of bronchodilator reversibility, shown by a growth of FEV1 more than 12% and 200 ml (or 12% of the predicted value in children) after inhaling a bronchodilator, or a rise in FEV1 of 12% and 200 ml (or a 20% increase in PEF on the same meter) after four weeks of anti-inflammatory treatment [3].

Children with asthma not only experience coughing, wheezing, difficulty breathing, and a feeling of chest constriction, especially during the night or early morning hours, but they also deal with a condition known as exercise-induced asthma. This phenomenon is common among children, largely due to their elevated levels of movement and exercise, which can lead to reduced tolerance for exercise and, consequently, a less active life style relative to their healthier peers [4]. Functional capacity impairment is frequently observed in children with bronchial asthma. As a result, assessing exercise capacity has become a crucial measure for determining functional limitations and evaluating the effectiveness of interventions. Tests conducted in the field, like shuttle tests (both walking and running) and 6 min walk test (6MWT), are regularly used to measure exercise capacity in children and adolescents with long-term respiratory issues, assisting in detecting any impairment in their physical performance [5].

Asthma results in mechanical overload of the respiratory musculature, causing both weakness and compensatory hypertrophy of the accessory inspiratory muscles. Elevated lung volumes lead to decreased lung compliance, contributing to fatigue in these muscles. This condition creates positive expiratory pleural pressures, which indicate ongoing contraction of the inspiratory muscles during expiration and may significantly impact their strength and efficiency. Moreover, individuals with asthma often breathe through their mouths, which can cause changes in cervical and spine posture, for example the tightness of the pectoral and cervical muscles and an anterior head position. This enhancement of forward head posture may lead to diminished maximum voluntary ventilation and maximum inspiratory pressure (MIP), along with reduced endurance in the deep neck flexor muscles [6].

Therefore this study was conducted to find whether there is a relation between pulmonary function, muscle endurance and functional capacity in children with bronchial asthma.

## Materials and methods

### Research design

This research is a cross sectional study.

### Sample size calculation

The sample size was calculated using the G*Power software (version 3.0.10). The correlation point biserial model was chosen. With a power of 0.80 (two-tailed), an alpha level of 0.05, and an effect size of 0.346 derived from a pilot study, the minimum adequate sample size is 60 participants.

### Subjects

**Fig. 1:**
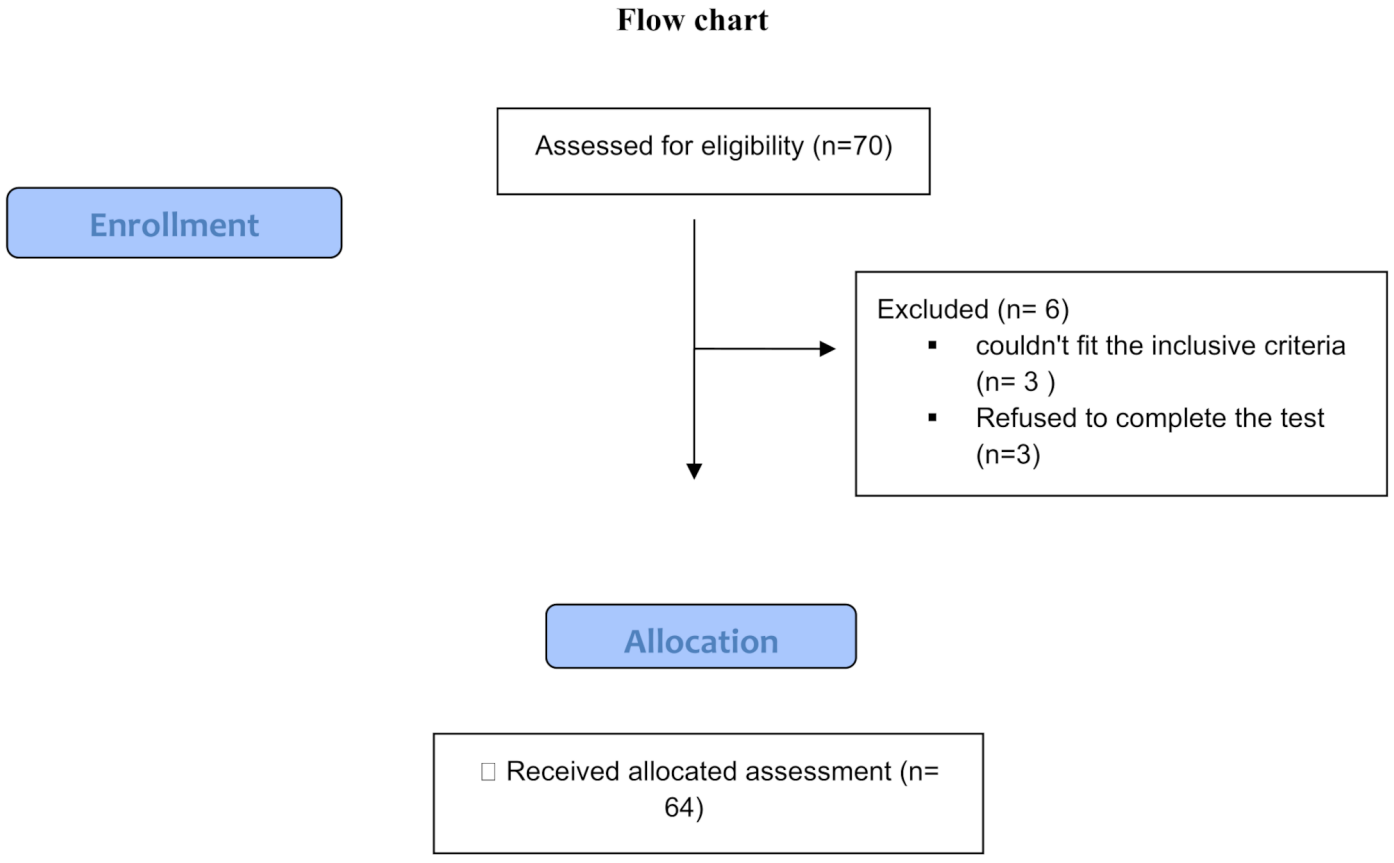
A flow chart showing the subjects recruitment and allocation

The study was conducted on sixty four pediatric patients with bronchial asthma (Fig. 1); they were selected from the outpatient clinic of the pediatric department of SadrEl-Abaseya Hospital from May 2024 to July 2024. The criteria for inclusion were as follows: children aged 8–10 years old, their body mass index (BMI) between 5th percentile to less than 85th percentile for age, gender and height according to world health organization (WHO) [7]. Children were diagnosed with bronchial asthma according to GINA guidelines as the following: mild (with the predicted forced expiratory volume in 1 s /forced vital capacity (FEV1/FVC) ≥ 80% and PEFR is between 20 and 30%), moderate (predicted FEV1/FVC range from 60 to 80% while PEFR range is > 30%), and severe (FEV1/FVC is ≤ 60 and PEFR is > 30%) [3].

Participants were eliminated if they exhibited one or more of the following conditions: significant vision or hearing loss, can’t walk independently because of lower limb injuries such as fractures, ankle sprain….etc., severe mental retardation, serious comorbidities, oxygen saturation (SpO2) below 94% (respiratory distress), cleft lip and palate deformities; abdominal or thoracic surgery within the last 3 weeks, or active hemoptysis.

### Procedures

The evaluation of the children took place during their routinely scheduled appointments. Each child was evaluated separately in a private, calm room. They were advised to skip large meals for at least two hours prior to the evaluation procedures.

### Evaluation of pulmonary function

Weight (in kilograms), height (in centimeters) and BMI (kg/m2) were recorded for every child before starting the evaluation procedures by standardized weight and height scale.

All children were evaluated by a portable spirometer ‘’Spirostik’’ introduced in Germany with a serial number (SN: 438751). The portable spirometer is a reliable and valid tool to screen and diagnose chronic airway diseases [8]. The essential parts of a spirometer are the mouth-piece, nose clip, tube, and an electronic apparatus for measuring airflow and computing spirometry parameters, which are displayed on a monitor [9].

According to **Can and Avci** [10], by using spirometery, each child was instructed to be in a comfortable sitting position, with the nose clip attached to the nose, and the mouth piece inserted inside the mouth and closed the lips completely around the piece to prevent air leakage. Each child was instructed to do the fastest and the deepest inspiration possible after 3 normal breaths, and then made a forced and rapid expiration for 6 s. VC, FEV1, PEF, and FEV1/FVC were recorded from the screen of the spirometer. The test took about 45 minutes to complete [11]. In this current study children with different severity of bronchial asthma were included (mild, moderate and severe).

### Evaluation of muscle endurance

Pressure bio-feedback (PBU) was utilized to evaluate the endurance of the deep cervical flexor muscles (DCFs) in the current study by applying the Cranio-cervical flexion test (CCFT).

Pressure biofeedback (PBU) demonstrates strong intra-rater and inter-rater reliability and validity [12]. It consists of a three-chamber, air-filled pressure cell (cuff), a catheter, and a sphygmomanometer gauge calibrated to 2 mmHg, with a measurement range of 0 to 200 mmHg [13].

The CCFT was conducted with the children in a crook-lying position. With the pressure biofeedback unit (PBU) positioned beneath the base of the skull and expanded to an initial level of 20 mmHg, the children were instructed to perform head nodding. The test comprised five gradual levels (22, 24, 26, 28, and 30 mmHg), requiring three repetitions at each level before advancing to the next. The test continued in this manner until either three ten-second holds at 30 mmHg were successfully completed or signs of compensatory activity in the sternocleidomastoid (SCM) or scalene muscles were observed or palpated. Other indicators for stopping included overshooting the target pressure, neck retraction, and flickering of the dial needle, as outlined by **Mohamed et al.** [14]

#### Evaluation of functional capacity

In the present study, functional capacity was evaluated using the 6-minute walk test (6MWT). The 6MWT is a commonly used exercise or stress evaluation method [15]. It is considered both valid and reliable for children with asthma, which increases its medical relevance [16]. This test is simple to administer, well accepted, and offers more accurate representation of daily activities and functional capacity than other walking assessments [17]. the evaluation was conducted on a flat, unobstructed 30-meter long corridor, allowing each child to wear their preferred footwear. Following the American Thoracic Society (ATS) guidelines [18], verbal instructions and encouragement were provided to all children while performing the test, with one statement permitted each minute. Prior to the start of the test, the children were notified that the objective was to cover as much distance as possible within six minutes. The evaluator walked behind each child to ensure safety without influencing their speed. Upon completion of the assessment, the distance covered by the participant in meters was documented, as referenced by **Abd El-Nabie et al.** [19]

### Statistical analysis

All statistical analyses were carried out utilizing SPSS software version 22. Associations between different pulmonary function parameters and both endurance and functional capacity were assessed through the computation of Spearman’s Rho coefficient (r_s_). Spearman’s Rho coefficient (r_s_) was analyzed according to subsequent criteria: r_s_ ≥ 0.8 very strong relationships; 0.6 ≤ r_s_< 0.8 strong relationships; 0.4 ≤ r_s_< 0.6 moderate relationship; 0.2 ≤ r_s_< 0.4 weak connections; r_s_< 0.2 very weak relationships. Mean ± standard deviation ($\bar x$± SD) and percentage expressed the data. The significance level for all statistical analyses was established at *P* < 0.05.

## Results

Sixty-four children participated in this research. Table 1 illustrates the participants’ features, including age, BMI, sex, and the asthma severity.

**Table 1:** Patient characteristics

*N* = 64\Features	$\bar x$±SD			
Age (years)	8.9 ± 0.8			
BMI (kg/m^2^)	18.2 ± 1.2			
Sex	Male		Female	
	33 (51.6%)		31 (48.4%)	
Asthma Severity	Mild	Moderate		Severe
	19 (29.7%)	29 (45.3%)		16 (25%)

$\bar x$: Mean, SD: standard deviation, BMI: body mass index

The means of the evaluation outcomes of the pulmonary function test were 74.2%, 70.14%, 81.45%, and 40% for the VC, FEV1, FEV1/FVC, and PEF, respectively. The findings of the endurance and functional capacity assessment indicated that the mean of the cervical flexion test was (26.06°) and 6MWT was 261.4 m (Table 2).

**Table 2:** Evaluation of the pulmonary function, endurance, and functional capacity of all patients

Variables	Minimum	Maximum	Mean	SD
VC (%)	41	105	74.2	13.5
FEV1 (%)	34	85	70.14	12.97
FEV1/FVC (%)	68	89	81.45	5.3
PEF (%)	13	67	40	13.1
Cervical flexion test (°)	20	30	26.06	3.67
6MWT (m)	115	420	261.4	101.4

SD: standard deviation, VC: vital capacity, L: liter, FEV1: forced expiratory volume in the first second, FEV1/FVC: ratio of the forced expiratory volume in the first second to the forced vital capacity, PEF: peak expiratory flow, °: degree, 6MWT: six-minute walking test, m: meter

**Fig. 2:**
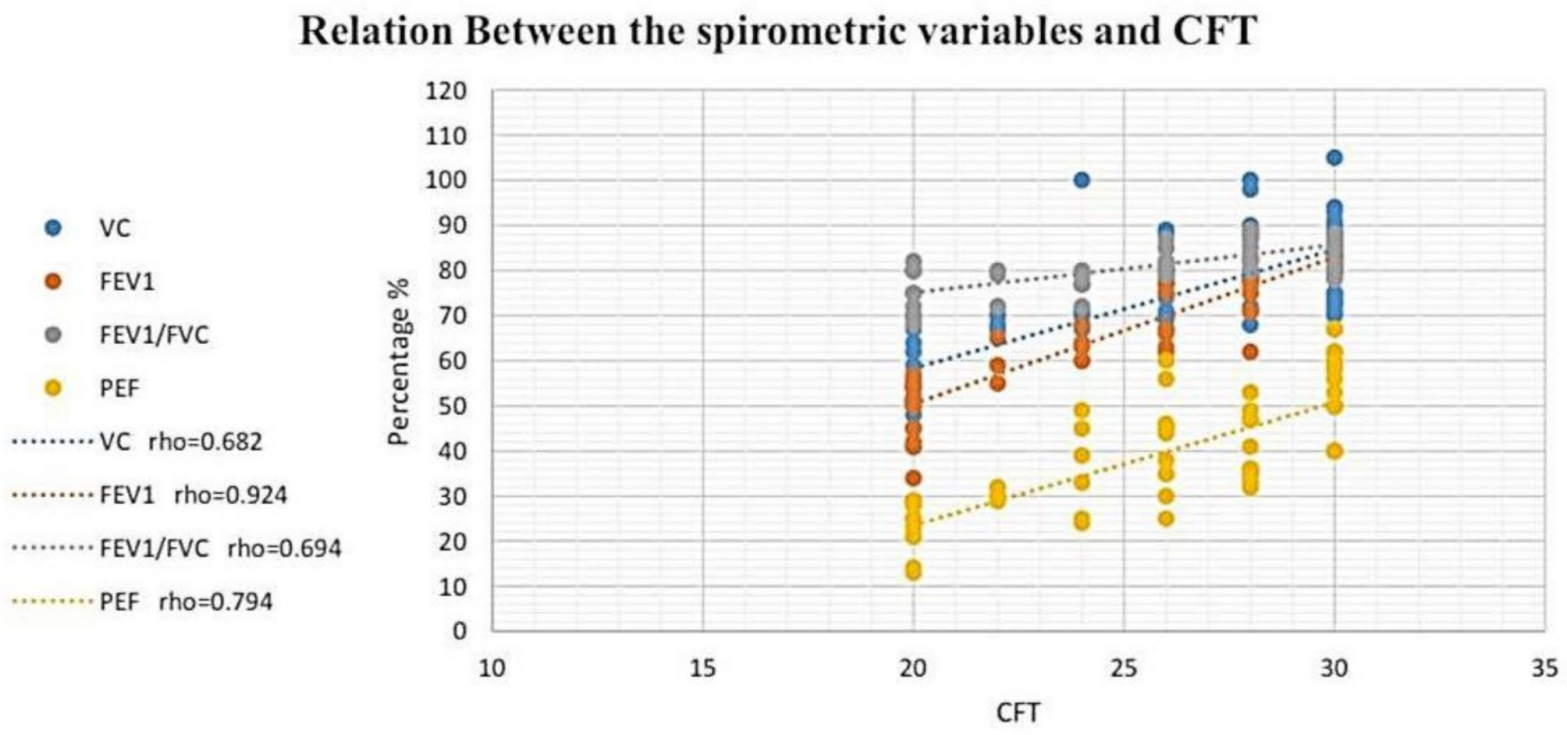
Relation between the spirometer variables and CFT

**Fig. 3:**
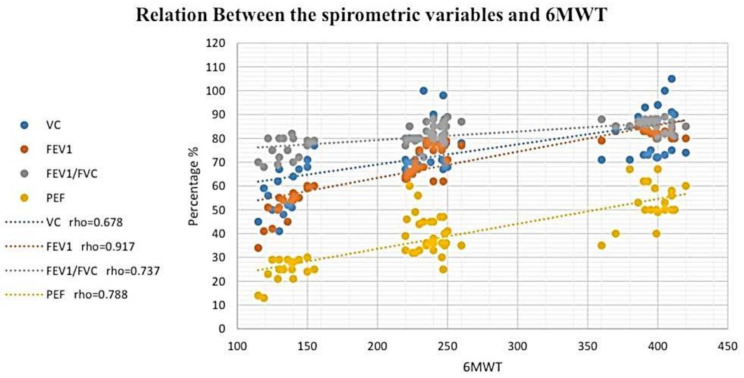
Relation between the spirometer variables and 6MWT

According to the study results (Fig 2 and 3), a significant and strong positive correlation existed between the VC and the results of the cervical flexion test. (*r*_s_ = 0.682, *P* ≤ 0.05) in addition to the 6MWT (*r*_s_ = 0.678, *P* ≤ 0.05). Moreover, there was a significant and very strong positive relationship between FEV1 and both cervical flexion test (*r*_s_ = 0.924, *P* ≤ 0.05) along with 6MWT (*r*_s_ = 0.917, *P* ≤ 0.05). Furthermore, a significant and strong positive connection was detected among FEV1/FVC and both the cervical flexion test (*r*_s_ = 0.694, *P* ≤ 0.05) as well as 6MWT (*r*_s_ = 0.737, *P* ≤ 0.05). Also, PEF was significantly, strongly, and positively connected with both cervical flexion test (*r*_s_ = 0.794, *P* ≤ 0.05) as well as 6MWT (*r*_s_ = 0.788, *P* ≤ 0.05) (Table 3).

**Table 3:** Relationships among pulmonary function, cervical endurance, and functional capacity in all patients

Variables	Cervical flexion test (°)	6MWT (m)
	*r*_s_	*r*_s_
VC (%)	0.682*	0.678*
FEV1 (%)	0.924*	0.917*
FEV1/FVC (%)	0.694*	0.737*
PEF (%)	0.794*	0.788*

VC: vital capacity, FEV1: forced expiratory volume in the first second, FEV1/FVC: ratio of the forced expiratory volume in the first second to the forced vital capacity, PEF: peak expiratory flow, °: degree, 6MWT: six-minute walking test, m: meter, *r*_*s*_: Spearman’s Rho correlation coefficients, *: correlation is significant at the 0.05 level

## Discussions

The objective of the present research was to assess the association between pulmonary function, muscle endurance, and functional capacity in pediatric patients suffering from bronchial asthma. The findings indicated that there was a strong positive correlation of pulmonary functions (FEV1, FEV1/FVC.PEFR.VC), muscle endurance and functional capacity.

The significant connection between asthma and physical activity is often overlooked and inadequately investigated. Physical activity can frequently trigger asthma-related symptoms, highlighting the condition’s nature or insufficient management. Evidence from population studies indicates that individuals with asthma generally have a diminished ability to participate in everyday activities and work, and they are less prone to participate in vigorous exercise compared to children who do not have asthma. Additionally, they are less likely to meet recommended levels of physical activity [20].

Considering the strong positive correlation of our study, the lack of muscle endurance (deep cervical flexor) may be the main cause of the deficiency of pulmonary functions. This was demonstrated by **Anwar et al.** [21]and **Tsimouris et al.** [22], who explained that weakness and insufficiency in the primary inspiratory muscles, particularly the diaphragm, in asthmatic children can lead to an overload of the accessory muscles, such as the sternocleidomastoid and scalenii. This condition may also result in weakness and decreased endurance of the deep cervical flexors. Additionally, it was demonstrated that factors such as hypo-mobility of the cervical spine, reduced neuromuscular endurance and strength in neck muscles, altered mental states, and diminished cervical proprioception can all impact respiratory function.

Improvement in the deep cervical flexor may improve the pulmonary function of the child. This is conducted by **Mohamed et al.** [14] and **Yu et al.** [23], who illustrated that significant improvements were observed not only in the cranio-cervical flexion test (CCFT), which assesses cervical endurance and peak expiratory flow rate (PEFR), but also in FEV1, FVC, and PEF following cervical stabilization exercises that involved stretching the superficial neck muscles and strengthening the deep cervical flexors in patients with anterior head posture.

Regarding the positive correlation of pulmonary function and functional capacity in our study and previous studies these results may be due to the fact that asthmatic children often experience limited physical exercise capacity primarily because of ventilatory constraints, muscle dysfunction, and cardiovascular issues. This indicates that poor pulmonary function adversely affects the functional capacity of asthmatic children. This was supported by **Yang et al.** [24] **.**Additionally, the findings of **Krishna et al.** [25]. align with ours, demonstrating that functional capacity positively correlates with FEV1%, FEV1 (in liters), FVC%, and FVC (in liters), in patients with COPD. However, the results by **Borgmann et al.** [26]reported that the total length walked in the 6-minute assessment (6MWT) by individuals with acute COPD consistently decreased over time, on the other hand FEV1 showed little change. Both COPD and bronchial asthma are classified as chronic obstructive lung diseases. Ultimately, we can confirm a statistically significant positive correlation between functional capacity and pulmonary functions, as demonstrated by **Murudkar** [27].

After showing the impact of muscle endurance and functional capacity on pulmonary function in children with bronchial asthma, I suggest that working on the endurance of cervical muscle especially deep cervical flexors will absolutely improve the child’s pulmonary function. Also working on functional capacity as advice the child for example to do swimming exercise will certainly improve the pulmonary function of the asthmatic child.

### Limitation

The current study was limited to detect the correlation of pulmonary function with muscle endurance and functional capacity in children with bronchial asthma aged from 8 to 10 years old. So, further studies are advised to detect this relation in children with bronchial asthma with different age group. Also, in the current study children needed additional time to be familiar and understand the procedures of evaluation clearly.

## Conclusion

According to the findings of this study, pulmonary function is positively correlated with muscle endurance and functional capacity, which may affect their capacity to perform the normal activities of daily living.

## Data Availability

The data supporting our findings are available from the corresponding author in reasonable request.
